# Higher Urinary Heavy Metal, Phthalate, and Arsenic but Not Parabens Concentrations in People with High Blood Pressure, U.S. NHANES, 2011–2012

**DOI:** 10.3390/ijerph110605989

**Published:** 2014-06-05

**Authors:** Ivy Shiue

**Affiliations:** 1 School of the Built Environment, Heriot-Watt University, Riccarton, EH14 4AS, Edinburgh, Scotland EH14 4AS, UK; E-Mail: I.shiue@hw.ac.uk; Tel.: + 44-131-451-4655; Fax: + 44-131-451-3161; 2 Owens Institute for Behavioral Research, University of Georgia, 215 S Jackson St., Athens, GA 30602, USA

**Keywords:** chemicals, environmental health, etiology, risk factor, hypertension

## Abstract

Link between environmental chemicals and human health has emerged but not been completely examined in risk factors. Therefore, it was aimed to study the relationships of different sets of urinary environmental chemical concentrations and risk of high blood pressure (BP) in a national, population-based study. Data were retrieved from United States National Health and Nutrition Examination Surveys, 2011–2012 including demographics, BP readings, and urinary environmental chemical concentrations. Analyses included chi-square test, t-test and survey-weighted logistic regression modeling. After full adjustment (adjusting for urinary creatinine, age, sex, ethnicity, and body mass index), urinary cesium (OR 1.56, 95%CI 1.11–2.20, P = 0.014), molybden (OR 1.46, 95%CI 1.06–2.01, P = 0.023), manganese (OR 1.42, 95%CI 1.09–1.86, P = 0.012), lead (OR 1.58, 95%CI 1.28–1.96, P < 0.001), tin (OR 1.44, 95%CI 1.25–1.66, P < 0.001), antimony (OR 1.39, 95%CI 1.10–1.77, P = 0.010), and tungsten (OR 1.49, 95%CI 1.25–1.77, P < 0.001) concentrations were observed to be associated with high BP. People with higher urinary mono-2-ethyl-5-carboxypentyl phthalate (OR 1.33, 95%CI 1.00–1.62, P = 0.006), mono-*n*-butyl phthalate (OR 1.35, 95%CI 1.13–1.62, P = 0.002), mono-2-ethyl-5-hydroxyhexyl (OR 1.25, 95%CI 1.05–1.49, P = 0.014), mono-*n*-methyl phthalate (OR 1.26, 95%CI 1.07–1.48, P = 0.007), mono-2-ethyl-5-oxohexyl (OR 1.25, 95%CI 1.07–1.48, P = 0.009), and monobenzyl phthalate (OR 1.40, 95%CI 1.15–1.69, P = 0.002) tended to have high BP as well. However, there are no clear associations between environmental parabens and high BP, nor between pesticides and high BP. In addition, trimethylarsine oxide (OR 2.47, 95%CI 1.27–4.81, P = 0.011) and dimethylarsonic acid concentrations (OR 1.42, 95%CI 1.12–1.79, P = 0.006) were seen to be associated with high BP. In sum, urinary heavy metal, phthalate, and arsenic concentrations were associated with high BP, although the causal effect cannot be established from the current study design. Elimination of environmental chemicals in humans would still need to be continued.

## 1. Introduction

The burden of high blood pressure (BP) has remained high in the U.S., affecting about one third of American adults in the current century [1]. The economic costs associated with hypertension are high for both individuals and the society. Exposure to environmental chemicals may induce atherosclerosis by increasing oxidative stressor or produce reactive oxygen species as superoxide ion, hydrogen peroxide, and hydroxyl radical, according to experimental research [2,3]. Previous epidemiological investigations were focusing on arterial disease, heart disease, and cardiovascular disease (CVD) as endpoints [4,5,6], but the relationship with BP, a strong risk contributor for many human chronic diseases as mentioned above in the pathway, is unclear. Previously, adverse intrauterine environments was found to be associated with increased risk of later CVD and obesity (*i.e.*, weight gain in animals at low doses) leading to population epidemic [7]. Recent animal models have shown that elevated offspring BP could be induced by maternal exposure to toxicants [8]. Experimental human research also have shown chemicals with estrogenic or endocrine disrupting activity and that exposure to these chemicals during critical stages of differentiation may have permanent long–lasting consequences while some of which may not be expressed or detected until later in life [9]. Following this context, therefore, it was aimed to examine the relationships of different sets of urine environmental chemical concentrations and risk of high BP in a national and population-based setting.

## 2. Experimental Section

### 2.1. Study Sample and Variables

As described elsewhere [10], United States National Health and Nutrition Examination Surveys (NHANES) has been a national, population-based, multi-year, cross-sectional study. Study sample are representative sample of the civilian, non-institutionalized US Population. In the current analysis, the 2011–2012 cohort as the most recent cohort was selected. Informed consents were obtained from participating subjects. The sampling method was a complex, multistage probability sampling design that was used to select a sample representative of the civilian non-institutionalized household population of the U.S. [11]. It started from selecting counties or small groups of contiguous counties and then went down to a block or group of blocks containing a cluster of households. After identifying eligible households within segments, individuals within households were invited for surveying [11]. Information on demographics, lifestyle factors, and self-reported medical conditions was obtained by household interview using questionnaires. BP was measured on all examinees 8 years and older at the household interview and for three times. In the present study, the second time BP measurement was used in the analysis. The standard measuring protocol can be found on the website under 2011–2012 study cohort [12]. In brief, participants with any of the following on both arms were excluded from the exam according to the standard protocol, rashes, gauze dressings, casts, edema, paralysis, tubes, open sores or wounds, withered arms, a-v shunts, and radical mastectomy or if BP cuff does not fit on the arm. For statistical analysis, people with ≥140 mmHg systolic BP and ≥90 mmHg diastolic BP were classified as high BP.

### 2.2. Biomonitoring

Urines were only collected in a smaller sample for about 20%–30% of the whole cohort [13], being representative, to measure environmental chemicals concentrations. Therefore, survey weights are to be applied in the statistical modelling. Urine specimens were processed, stored and shipped to Division of Laboratory Sciences, National Center for Environmental Health, National Centers for Disease Control and Prevention, Atlanta, Georgia. Liquid samples of are introduced into the Inductively coupled plasma-mass spectrometry, a multi-element analytical technique, through a nebulizer and spray chamber carried by a flowing argon stream to detect heavy metals [14,15]. High performance liquid chromatography-electrospray ionization-tandem mass spectrometry was used for the quantitative detection in urine of phthalate metabolites [16,17]. A sensitive method for measuring BPA, BP-3, triclosan, parabens, and pesticides was developed to use on–line solid phase extraction coupled to HPLC and tandem mass spectrometry [18,19]. With the use of isotopically labeled internal standards, the detection limits in 100 μL of urine are 0.1–2.3 nanograms per milliliter (ng/mL), sufficient for measuring urinary levels of phenols in non-occupationally exposed subjects. Arsenic is measured through the use of inductively coupled-plasma dynamic reaction cell-mass spectrometry.

### 2.3. Statistical Analysis

Adults aged 20 and above were included in the analysis. As urinary environmental chemicals concentrations were highly right skewed, they were all log transformed in the analyses. Effects of urinary environmental chemical concentrations on risk of high BP were examined by *t*-test and logistic regression model, with P < 0.05 considered statistically significant. Covariates including urinary creatinine, age, sex, ethnicity, and body mass index (BMI) [20] were adjusted. Models were also weighted for the survey design. Statistical software STATA version 13.0 (STATA, College Station, TX, USA) was used to perform all the analyses. Since the present study was only a secondary data analysis, no further ethics approval was required.

## 3. Results and Discussion

The study cohort in 2011–2012 contained 9,756 participants with 3,035 people being classified as high BP (31.1%). Table 1 presents the characteristics of included participants. About 20% were identified as overweight (BMI 25–30) while 33% were classified as obese (BMI > 30). Table 2 shows the associations of urinary heavy metal concentrations on risk of high BP. After full adjustments (including urinary creatinine, age, sex, ethnicity, and BMI) and subsample weighting (following the biomonitoring examination in a small but representative proportion, 20%–30%, of the study population), urinary cesium (OR 1.56, 95%CI 1.11–2.20, P = 0.014), molybden (OR 1.46, 95%CI 1.06–2.01, P = 0.023), manganese (OR 1.42, 95%CI 1.09–1.86, P = 0.012), lead (OR 1.58, 95%CI 1.28–1.96, P < 0.001), tin (OR 1.44, 95%CI 1.25–1.66, P < 0.001), antimony (OR 1.39, 95%CI 1.10–1.77, P = 0.010), and tungsten (OR 1.49, 95%CI 1.25–1.77, P < 0.001) concentrations were observed to be associated with high BP.

**Table 1:** Characteristics of participants (N = 9,756).

	N (%) or Mean ± SD
*Age*	*31.4 ± 24.6*
<18	3,892 (39.9%)
18–39	2,261 (23.2%)
40–79	3,240 (33.2%)
80	363 (3.7%)
*Sex*	
Male	4,856 (49.8%)
Female	4,900 (50.2%)
*Ethnicity*	
Mexican American	1,355 (13.9%)
Other Hispanic	1,076 (11.0%)
Non-Hispanic white	2,973 (30.5%)
Non-Hispanic black	2,683 (27.5%)
Mixed/other	1,669 (17.1%)
High blood pressure *	*3,035 (31.1%)*
Systolic blood pressure	118.7 ± 18.6
Diastolic blood pressure	66.3 ± 16.1
*Body mass index*	*25.3 ± 7.7*
<18.5	1,833 (18.8%)
18.5–24.9	2,669 (27.4%)
25.0–29.9	2,019 (20.7%)
≥30.0	3,235 (33.2%)

Note: * High blood pressure denotes systolic blood pressure ≥ 140 mmHg and diastolic blood pressure ≥ 90 mmHg.

**Table 2:** Associations between heavy metals and high blood pressure.

	Normal BP (n = 2,193)	High BP (n = 314)	P value	Adjusted Model *	P value	Weighted Model *	P value
Mercury	0.62 ± 1.45	0.67 ± 1.83	0.578	1.13 (1.00–1.29)	**0.056**	1.06 (0.89–1.26)	0.528
Barium	1.85 ± 2.77	1.76 ± 1.81	0.562	1.01 (0.88–1.16)	0.871	1.15 (0.91–1.44)	0.220
Cadmium	0.30 ± 0.43	0.26 ± 0.51	0.199	1.06 (0.89–1.27)	0.504	1.02 (0.83–1.87)	0.832
Cobalt	0.49 ± 0.86	0.54 ± 0.88	0.318	**1.28 (1.07–1.52)**	**0.006**	1.35 (0.97–1.88)	0.073
Cesium	4.78 ± 3.17	5,11 ± 3.16	0.086	**1.74 (1.38–2.21)**	**<0.001**	**1.56 (1.11–2.20)**	**0.014**
Molybdenum	59.90 ± 57.00	71.64 ± 70.21	0.001	**1.49 (1.26–1.77)**	**<0.001**	**1.46 (1.06–2.01)**	**0.023**
Manganese	0.17 ± 0.44	0.19 ± 0.20	0.629	**1.40 (1.18–1.66)**	**<0.001**	**1.42 (1.09–1.86)**	**0.012**
Lead	0.59 ± 1.05	0.69 ± 1.00	0.112	**1.62 (1.38–1.91)**	**<0.001**	**1.58 (1.28–1.96)**	**<0.001**
Tin	1.47 ± 3.17	2.18 ± 4.03	0.0004	**1.45 (1.30–1.63)**	**<0.001**	**1.44 (1.25–1.66)**	**<0.001**
Antimony	0.08 ± 0.12	0.10 ± 0.17	0.006	**1.56 (1.29–1.89)**	**<0.001**	**1.39 (1.10–1.77)**	**0.010**
Strontium	121.20 ± 121.08	115.82 ± 100.78	0.454	0.99 (0.85–1.15)	0.904	1.01 (0.71–1.43)	0.967
Thallium	0.20 ± 0.14	0.21 ± 0.16	0.272	**1.34 (1.09–1.66)**	**0.006**	1.16 (0.84–1.62)	0.350
Tungsten	0.16 ± 0.75	0.18 ± 0.24	0.636	**1.39 (1.21–1.60)**	**<0.001**	**1.49 (1.25–1.77)**	**<0.001**
Uranium	0.01 ± 0.07	0.01 ± 0.03	0.817	**1.19 (1.02–1.38)**	**0.024**	1.08 (0.89–1.30)	0.433

Note: * Adjusted for urine creatinine, age, sex, ethnicity, and body mass index in the adjusted model, and additionally adjusted for survey weighting in the weighted model.

In Table 3, associations between industry-associated chemicals and high BP are presented. People with higher urinary mono-2-ethyl-5-carboxypentyl phthalate (OR 1.33, 95%CI 1.00–1.62, P = 0.006), mono-*n*-butyl phthalate (OR 1.35, 95%CI 1.13–1.62, P = 0.002), mono-2-ethyl-5-hydroxyhexyl (OR 1.25, 95%CI 1.05–1.49, P = 0.014), mono–*n*–methyl phthalate (OR 1.26, 95%CI 1.07–1.48, P = 0.007), mono-2-ethyl-5-oxohexyl (OR 1.25, 95%CI 1.07–1.48, P = 0.009), and monobenzyl phthalate (OR 1.40, 95%CI 1.15–1.69, P = 0.002) tended to have high BP. However, there were no clear associations between environmental parabens and high BP, nor between pesticides and high BP. In addition, urinary trimethylarsine oxide (OR 2.47, 95%CI 1.27–4.81, P = 0.011) and dimethylarsonic acid concentrations (OR 1.42, 95%CI 1.12–1.79, P = 0.006) were also seen to be associated with high BP (see Table 4).

### 3.1. Main Findings

In the present national, population-based, cross-sectional study, the relationships of different sets of urinary environmental chemicals concentrations and the risk of high BP were examined. It was observed that higher urinary cesium, molybden, manganese, lead, tin, antimony, tungsten, mono-2-ethyl-5-carboxypentyl phthalate, mono-*n*-butyl phthalate, mono-2-ethyl-5-hydroxyhexyl, mono-*n*-methyl phthalate, mono-2-ethyl-5-oxohexyl, monobenzyl phthalate, trimethylarsine oxide, and dimethylarsonic acid concentrations were associated with high BP. However, there are no clear associations between environmental parabens and high BP, nor between pesticides and high BP. There were a few more significant associations after covariates adjustments. However, those significant associations disappeared (such as certain heavy metals, certain phthalate metabolites, and certain arsenic concentrations) after additionally adjusting for subsample weighting, implying the failure to generalize those potential significant associations to the whole U.S. population.

**Table 3:** Associations between industry–associated chemicals and high blood pressure.

	Normal BP (n = 2180)	High BP (n = 309)	P value	Adjusted model *	P value	Weighted model *	P value
Mono(carboxynonyl) phthalate	4.81 ± 16.80	6.28 ± 24.14	0.178	**1.27 (1.12–1.45)**	**<0.001**	1.19 (0.98–1.45)	0.073
Mono(carboxyoctyl) phthalate	51.35 ± 111.28	51.66 ± 115.20	0.964	1.06 (0.96–1.18)	0.239	1.03 (0.84–1.27)	0.730
Mono-2-ethyl-5-carboxypentyl phthalate	26.93 ± 62.83	27.51 ± 29.98	0.871	**1.37 (1.20–1.57)**	**<0.001**	**1.33 (1.10–1.62)**	**0.006**
Mono-*n*-butyl phthalate	23.58 ± 87.67	25.47 ± 40.33	0.709	**1.32 (1.19–1.48)**	**<0.001**	**1.35 (1.13–1.62)**	**0.002**
Mono-(3-carboxypropyl) phthalate	12.62 ± 84.15	12.50 ± 57.81	0.981	**1.19 (1.08–1.31)**	**<0.001**	1.13 (0.98–1.30)	0.080
Mono-ethyl phthalate	183.80 ± 832.54	141.27 ± 435.73	0.379	1.09 (0.99–1.19)	0.074	1.14 (0.97–1.34)	0.104
Mono-(2-ethyl-5-hydroxyhexyl)	18.26 ± 50.68	17.40 ± 23.11	0.769	**1.30 (1.15–1.47)**	**<0.001**	**1.25 (1.05–1.49)**	**0.014**
Mono-(2-ethyl)-hexyl phthalate	3.40 ± 8.41	2.69 ± 3.76	0.143	1.04 (0.92–1.18)	0.544	1.05 (0.82–1.33)	0.699
Mono-*n*-methyl phthalate	4.39 ± 22.85	10.80 ± 75.81	0.002	**1.24 (1.13–1.37)**	**<0.001**	**1.26 (1.07–1.48)**	**0.007**
Mono-isononyl phthalate	4.30 ± 13.70	4.23 ± 15.20	0.937	1.00 (0.90–1.10)	0.957	1.01 (0.87–1.16)	0.942
Mono-(2-ethyl-5-oxohexyl)	11.24 ± 26.39	11.29 ± 14.98	0.974	**1.32 (1.16–1.50)**	**<0.001**	**1.25 (1.07–1.48)**	**0.009**
Mono-benzyl phthalate	11.21 ± 19.74	16.91 ± 29.84	<0.001	**1.37 (1.21–1.54)**	**<0.001**	**1.40 (1.15–1.69)**	**0.002**
Mono-isobutyl pthalate	13.13 ± 22.17	15.71 ± 25.15	0.060	**1.27 (1.11–1.44)**	**<0.001**	1.14 (0.92–1.41)	0.213
Benzophenone-3	287.97 ± 2,063.09	183.41 ± 724.58	0.377	0.96 (0.90–1.02)	0.167	0.99 (0.88–1.11)	0.794
Bisphenol A	3.18 ± 8.47	3.14 ± 7.91	0.936	1.11 (0.96–1.27)	0.149	0.94 (0.74–1.20)	0.615
Triclosan	90.74 ± 272.57	71.09 ± 235.00	0.228	0.96 (0.90–1.03)	0.308	0.96 (0.87–1.06)	0.377
Butyl paraben	2.01 ± 13.14	1.51 ± 7.36	0.508	1.02 (0.92–1.12)	0.728	1.06 (0.90–1.25)	0.467
Ethyl paraben	15.51 ± 60.47	8.77 ± 22.60	0.053	1.01 (0.93–1.10)	0.771	0.99 (0.87–1.12)	0.846
Methyl paraben	214.79 ± 482.94	279.78 ± 979.54	0.060	**1.08 (1.00–1.16)**	**0.045**	1.05 (0.92–1.20)	0.457
Propyl paraben	50.64 ± 145.75	58.85 ± 237.71	0.399	1.04 (0.98–1.11)	0.181	1.04 (0.95–1.14)	0.329

Note: * Adjusted for urine creatinine, age, sex, ethnicity, and body mass index in the adjusted model, and additionally adjusted for survey weighting in the weighted model.

**Table 4:** Associations between pesticide and arsenic and high blood pressure.

	Normal BP (n = 2,478)	High BP (n = 387)	P value	Adjusted model *	P value	Weighted model *	P value
2,5-Dichlorophenol	148.62 ± 1,009.99	148.71 ± 1,215.77	0.999	1.02 (0.96–1.08)	0.549	0.96 (0.89–1.04)	0.299
2,4-Dichlorophenol	4.61 ± 29.06	4.46 ± 29.11	0.931	1.08 (0.99–1.19)	0.098	1.02 (0.88–1.19)	0.770
Total arsenic	19.38 ± 57.14	17.59 ± 31.88	0.557	**1.11 (1.00–1.23)**	**0.046**	1.13 (0.99–1.29)	0.066
Arsenous acid	0.54 ± 1.33	0.51 ± 0.35	0.687	0.97 (0.75–1.25)	0.819	0.97 (0.66–1.43)	0.886
Arsenic acid	0.66 ± 0.83	0.64 ± 0.19	0.683	0.82 (0.38–1.74)	0.602	0.47 (0.16–1.41)	0.164
Arsenobetaine	11.24 ± 51.33	9.13 ± 24.00	0.434	1.00 (0.92–1.08)	0.921	0.97 (0.86–1.10)	0.671
Arsenocholine	0.25 ± 0.53	0.21 ± 0.08	0.128	0 58 (0.31–1.05)	0.073	0.35 (0.17–0.74)	0.009
Dimethylarsonic acid	5.97 ± 7.83	6.42 ± 8.87	0.304	**1.28 (1.11–1.50)**	**0.001**	**1.42 (1.12–1.79)**	**0.006**
Monomethylarsonic acid	0.90 ± 1.51	0.87 ± 0.53	0.727	1.16 (0.87–1.55)	0.316	1.40 (0.80–2.46)	0.223
Trimethylarsine oxide	0.23 ± 1.51	0.21 ± 0.17	0.818	1.32 (0.96–1.83)	0.088	**2.47 (1.27–4.81)**	**0.011**

Note, * Adjusted for urine creatinine, age, sex, ethnicity, and body mass index in the adjusted model, and additionally adjusted for survey weighting in the weighted model.

### 3.2. Possible Mechanisms

Cesium was previously found in people exposed to Chernobyl radiation [21], and animal models in rats, dogs, rabbits, and *in vivo* have also observed that cardiovascular system or coronary blood flow could impair after long contamination of cesium in drinking water [22,23,24,25,26]. Effect of manganese on hypertension is not a new discovery. Since 1950s, there have been lots of research addressing the relationship. Lead could cause enhanced B cell activities and impairs host resistance to several bacterial and viral infections and can differentially modifies cytokine production *in vitro* and *in vivo* [27]. Lead exposure was found to result in a marked elevation of BP, a significant reduction in urinary NO metabolites (NO(chi)) excretion, and up–regulations of endothelial and inducible NOS abundance in the kidney (which could impact filtration rates and normalization using creatinine), aorta, and heart and of neuronal NOS in the cerebral cortex and brain stem in animals [28]. There have also been some literature on tin as a toxic heavy metal. The source could be from canned foods and the absorption of iron could be diminished when tin present [29]. How antimony could be related to risk of high blood pressure is inconclusive, although how it could affect the cardiovascular system started in the 1960s [30]. Tungsten is thrombogenic and proinflammatory, but its toxicity and carcinogenicity on cardiovascular health is not well examined [31,32]. Tungsten coils were prevalent in the clinical use for the occlusion of intracranial aneurysms, varicocele veins, and other abnormal vascular connections [33]. Therefore, people with intracranial aneurysms after the treatment may experience higher tungsten volumes in the body than people without. In the subsequent analysis, after additionally excluding people with ever stroke (n = 227), its effect on risk of high BP has remained significant (data not shown). Antimony has long been related to pneumoconiosis and dermatitis (acute effect) [34] and previously it was also found to be correlated with cardiovascular endpoints in smelter workers and gastrointestinal disorders (chronic effect) by inhalation [35,36,37].

Phthalates and bisphenol A, two chemical estrogens widely used in the food packaging industry, leach from the polymers into food and water under normal conditions [38] and can be detected in human urine. They can migrate out of the plastic product and into the environment and are suspected to act as hormone mimics (and endocrine-disrupting compounds) [39,40]. Animal studies have shown that chronic exposure to these, even at low dose, can alter some biological endpoints [41,42]. Other evidence further showed that mono-butyl phthalate disturbs the glycolytic pathway and can suppress other proteins that are involved in DNA transcription, RNA biogenesis and protein synthesis [6]. These have hypothesized to contribute to cardiovascular disease pathway and highlighted the need for new disease prevention by eliminating these potential risks. In recent meta–analyses, the pooled effect estimates of arsenic concentrations were found to be from 1.19 (95%CI 1.2–3.0) to 1.27 (95%CI 1.09–1.47) [43,44]. The former wasfrom a meta–analysis by searching Ovid, EMBASE, MEDLINE and Google Scholar with literature up to February 2012 while the latter was from a systematic review by searching PubMed, Embase, and TOXLINE between January 1966 through March 2011 with no language restrictions. In the present study, although the total arsenic concentrations were not significantly associated with risk of high BP, dimethylarsonic acid and trimethylarsine oxide concentrations were observed to be related to an increased risk of high BP which is similar to a previous study using NHANES 2003–2008 data [45]. Animal studies have also suggested that the chemical propensity of arsenic to oxidize vicinal thiols could potentially affect a number of cellular proteins with reactive thiols including endothelial NO synthase [46].

### 3.3. Strengths and Limitations

There are a few strengths and limitations worthy of discussion. First, this study was conducted in a large and nationally representative human sample with mixed ethnicities. Moreover, different sets of chemicals were able to be included for examination and heavy metals such as cesium , manganese, tin, strontium, and Thallium that had not been examined were included in this study cohort. However, there could be still other emerging chemicals from the environment that we might not know and would need future research to identify and examine. The causality effect cannot be established in the present study due to the cross-sectional study design in nature. Future studies with a longitudinal study design to confirm or refute the current findings, if at all, and to understand the persisting risk effects along the life course from those mentioned above environmental chemicals would be suggested.

## 4. Conclusions

In sum, urinary environmental chemical concentrations such as cesium, molybden, manganese, lead, tin, antimony, tungsten, phthalates, and arsenic were associated with high BP. It is known that environmental chemicals present in air, food, water, and soil, but the combination of epigenetic effects and gene induction is still less known [47]. Elimination of environmental chemicals in humans would still need to be continued in the coming decades and prevention strategies to shift the focus from individual level to social or national level might need to be considered as to better manage environments to reduce exposure to these modifiable risks.
